# Comparing Auditory Noise Treatment with Stimulant Medication on Cognitive Task Performance in Children with Attention Deficit Hyperactivity Disorder: Results from a Pilot Study

**DOI:** 10.3389/fpsyg.2016.01331

**Published:** 2016-09-05

**Authors:** Göran B. W. Söderlund, Christer Björk, Peik Gustafsson

**Affiliations:** ^1^Faculty of Teacher Education and Sport, Sogn og Fjordane University CollegeSogndal, Norway; ^2^Department of Pupil Welfare, Municipality of SkellefteåSkellefteå, Sweden; ^3^Child and Adolescent Psychiatry, Department of Clinical Sciences, Lund UniversityLund, Sweden

**Keywords:** ADHD, white noise, stimulant medication, cognitive performance, stochastic resonance

## Abstract

**Background:** Recent research has shown that acoustic white noise (80 dB) can improve task performance in people with attention deficits and/or Attention Deficit Hyperactivity Disorder (ADHD). This is attributed to the phenomenon of stochastic resonance in which a certain amount of noise can improve performance in a brain that is not working at its optimum. We compare here the effect of noise exposure with the effect of stimulant medication on cognitive task performance in ADHD. The aim of the present study was to compare the effects of auditory noise exposure with stimulant medication for ADHD children on a cognitive test battery. A group of typically developed children (TDC) took the same tests as a comparison.

**Methods:** Twenty children with ADHD of combined or inattentive subtypes and twenty TDC matched for age and gender performed three different tests (word recall, spanboard and n-back task) during exposure to white noise (80 dB) and in a silent condition. The ADHD children were tested with and without central stimulant medication.

**Results:** In the spanboard- and the word recall tasks, but not in the 2-back task, white noise exposure led to significant improvements for both non-medicated and medicated ADHD children. No significant effects of medication were found on any of the three tasks.

**Conclusion:** This pilot study shows that exposure to white noise resulted in a task improvement that was larger than the one with stimulant medication thus opening up the possibility of using auditory noise as an alternative, non-pharmacological treatment of cognitive ADHD symptoms.

## Background

Attention Deficit Hyperactivity Disorder (ADHD) is one of the most common psychiatric disorders worldwide and prevalence estimates range from 3 to 12% (Paule et al., 2000; Biederman and Faraone, 2005; Polanczyk et al., 2014). These estimates differ with age: 6–9% in the youth population and 3–5% in the adult population (Froehlich et al., 2007; Dopheide and Pliszka, 2009). Attention deficits in ADHD comprise difficulties in sustaining attention and following instructions as well as being seemingly inattentive when spoken to directly, while hyperactivity is manifested by overactivity, restlessness and impulsivity (APA, 2013). Children with attention deficits display problems in working memory, particularly in auditory working memory, as they seem to have a listening problem and they need auditory information to be repeated (Alderson et al., 2015; Söderlund and Nilsson Jobs, 2016). Moreover, ADHD diagnosed children also display significant deficits in verbal- and visuo- spatial working memory (Alderson et al., 2013; Gau and Chiang, 2013). The ability to keep verbally given instructions in mind and follow them is important for schoolwork and ADHD is therefore commonly associated with school failures and academic under-achievement (Faraone et al., 1993; Barkley et al., 2006; Serra-Pinheiro et al., 2008).

Stimulant medication, e.g., methylphenidate, can be used to treat behavioral problems in ADHD and can help to improve school performance (Evans et al., 2001; Greenhill et al., 2002; Scheﬄer et al., 2009; Wigal et al., 2011). Methylphenidate is shown to increase extracellular dopamine in the brain through blockade of dopamine transporters, which in turn amplifies weak dopamine signals and thus increases the signal-to-noise ratio enhancing the salience of the target task (Volkow et al., 2001). When task salience increases, this improves motivation, attention and thus performance in, e.g., mathematical tasks (Volkow et al., 2004). However, the best dose for optimal cognitive functioning was found to be lower than the best dose for school behavior (Hale et al., 2011). In addition, it is not evident that stimulant medication improves learning processes (Molina et al., 2009; Hellwig-Brida et al., 2011). Interestingly, the uncertain effects of stimulant medication on academic achievement have long since been reported (Adams, 1982). There are also concerns regarding the potential for drug abuse (Gordon et al., 2004), the long term duration of treatment effects (MTA Cooperative Group, 2004), and the possible effects of stimulant drugs on the developing brain (Andersen, 2005). Furthermore, at least one third of children have been found to discontinue their medical treatment after 2 years (Bussing et al., 2005), sometimes because of an unsatisfactory effect of the treatment but probably also because of poor motivation and a feeling of stigmatization by having to take a medicine. Although positive effects of medication are found over the age span, side effects are more prominent among children (Cornforth et al., 2010). All the above-enumerated uncertainties about medication underscore the urge for finding non-pharmacological alternative treatments of ADHD symptoms, in particular to improve school performance for children that are at risk of failures.

Normally, noise has a detrimental effect on all kinds of cognitive performance but recent research has shown prominent effects of white noise exposure on various cognitive tasks in children with attention deficits (Söderlund et al., 2010; Helps et al., 2014) and children with ADHD (Söderlund et al., 2007; Baijot et al., 2016). Noise benefit is not exclusively for children; positive noise effects are also found among inattentive adults (Söderlund et al., 2009; Flodin et al., 2012). It is suggested that this noise benefit is caused by the phenomenon of stochastic resonance (SR) in which a certain amount of noise can facilitate signal transmission in the brain, and increase the signal-to-noise ratio, and thus the performance on various tasks (McDonnell and Ward, 2011). The link between attention and noise benefit is explained in the Moderate Brain Arousal Model (MBA) that postulates that brains with low levels of internal neural noise, as in ADHD, require more external noise to work at optimum level (Sikström and Söderlund, 2007). The MBA model assumes that noise either regulates dopamine transmission or substitutes its effects on neural communication. Dopamine modulates the neural cells’ responses to the environment and determines the probability that a neuron will fire following the presentation of a stimulus, i.e., the neural cells’ gain parameter (Servan-Schreiber et al., 1990). Alterations in dopamine function are related to individual differences in attention (Prince, 2008), cognition (Braver and Barch, 2002), and motivated behavior (Grace et al., 2007). The MBA model suggests further that a hypodopaminergic brain, e.g., the brain of a child with an ADHD diagnosis, needs higher input of noise to function at its full potential, due to a low gain parameter owing to low levels of neural noise in the brain as a result of deficient dopamine levels. This implies that more external environmental noise is required for optimal performance in cognitive tasks for ADHD children (low gain) compared to normally developed children with a high gain (Sikström and Söderlund, 2007). If noise therapy is in parity with stimulant medication, noise exposure could be an interesting non-pharmacological treatment of ADHD.

The aim of the present pilot study was to compare the effects of auditory noise and stimulant medication on cognitive task performance in a group of children diagnosed with ADHD. A group of typically developed children (TDC) took the same tests in noise versus silent condition as a comparison. This is the first time, to our knowledge, that white noise exposure is compared with stimulant medication on a cognitive test battery in a group of children diagnosed with ADHD.

In this paper we study the effects of white noise exposure and stimulant medication on a cognitive test battery consisting of three different tasks: (i) a verbal episodic memory task; (ii) a visuo-spatial working memory task; and (iii) a verbal 2-back task. The group of TD children took all three tests in noise vs. silent conditions. Both the verbal- and the visuo-spatial task have shown substantial effects of noise in earlier studies (Söderlund et al., 2007, 2010; Helps et al., 2014). The verbal 2-back task is an extension of earlier studies from our research group; the n-back task is often used in the ADHD context as it tests different aspects of executive functioning like vigilance and working memory processing, it demands continuous updating and it is sensitive to stimulant medication (Kobel et al., 2009; Strand et al., 2012). Our specific predictions are as follows: (i) overall we expect TD children to have a performance superior to the ADHD group in all tasks; (ii) noise will improve performance for the ADHD group whereas it will disrupt performance for the TDC group; (iii) medication will improve performance for the ADHD group. Regarding possible interactions between noise and medication we have no firm predictions as noise may either improve performance for medicated children, or may instead push them over the top and be detrimental to performance.

## Materials and Methods

### Participants and Recruitment

Ethical approval was obtained from the Ethical Review Board in Stockholm (EPN 2008/1744 -31). Written consent was obtained from the headmasters of all participating schools and from parents of participating children. All participating children gave oral approval. Prior to the start of the study, parents were sent information forms and were given the possibility to opt their children out of the study at any time without giving reason. For the TDC group headmasters at four schools in the municipality of Skellefteå approached participating children’s parents for approval. The children with an ADHD diagnosis were informed and contacted by the National Association of Attention and the parents that volunteered to participate sent written consent to the research leader.

First, the twenty children in the ADHD group were selected. Sample size and power calculations were based on effect sizes from prior studies, η^2^ = 0.15 -0.39 (Söderlund et al., 2007, 2010; Söderlund and Sikström, 2012). Diagnoses in the ADHD group were set from 6 to 30 months ahead of the present study by the child and adolescent psychiatry department in the municipality of Skellefteå. All participants in the ADHD group were diagnosed as having ADHD, 13 with combined type (ADHD-C) and 7 with predominantly inattentive type (ADHD-I). Inattentivness is, according to prior studies, the crucial factor to yield noise benefit. All participants were medicated with methylphenidate and adapted to medication at the time of the study. There were no dropouts in the ADHD group; all twenty approached participants completed both test occasions. See also **Table 1** for description of participants.

**Table 1:** Participants’ characteristics: Teachers’ assessments of school performance, of inattention, and hyperactivity on the SNAP score.

Diagnosis	Boy/Girl	Age (SD)	School performance 1: above, 2: average, 3:below	Hyperactivity	Attention	Total (H + A)
ADHD	16/4	12.9 (2.3)	2.1 (0.8)	14.2 (5.0)	15.9 (5.0)	29.6 (8.4)
ADHD-C	13/0	12,9	2.2	17.3 (4.49	17.6 (4.4)	33.9 (6.5)
ADHD-I	3/4	12,9	2.0	9.4 (3.4)	13.3 (5.0)	22.7 (6.4)
Control	11/9	13.9 (1.3)	1.7 (0.7)	0.60 (0.9)	2.5 (3.4)	2.9 (4.2)
ADHD vs. Control *p*-value		0.11 (ns)	0.067 (ns)	<0.0005	<0.0005	<0.0005
ADHD-I vs. ADHD-C *p*-value				<0.0005	0.079	0.003

All of the twenty TDCs were screened according to the SNAP rating scale (Swanson et al., 2007). The SNAP score makes ratings between 0 and 3 on 18 questions that follow the DSM-5 criteria closely (APA, 2013); the 0 and 1 rating are considered as normal scores. Participants that scored low were assigned to the TDC group (below 5 p in all; the score for an ADHD diagnosis is between 36 and 54 p). Moreover, the 20 TDC children were also selected and matched with the ADHD group by age and gender. This screening took place in eight different classes in order to get a group of 20 participants that diverged significantly from the experiment group in only ADHD related symptoms, i.e., attention and hyperactivity. Teachers set the SNAP scores the week before or after the data collection, both for all groups. To control intellectual ability, teachers made an evaluation of all children’s school performance on three levels: average, above average, and below average to control that groups were comparable in this aspect (Söderlund et al., 2007). Children assessed by teachers as average or over average in cognitive function were included in the TDC group. There were no dropouts in the TDC group; all twenty participants selected after the screening took both tests.

### Procedure and Test Battery

All experiments were conducted at the participants’ schools on two different occasions. The three experiments were programmed in E-prime (Psychology software) and presented on a 15′ lap top computer screen. Instructions were given in writing on the screen. Participants sat on a comfortable chair about 70 cm away from the screen. ADHD children and TDC conducted all tasks in either absence or presence of white noise on each occasion. In the noise condition the noise level was set to 80 dB in accordance with findings from earlier studies where noise effects were obtained (Söderlund et al., 2007, 2010) and was delivered binaurally through high quality headphones. The three different tasks were given in the same order as seen below on each test and repeated for 2 days. The delay between the two test varied from 3 to 6 days. The ADHD group received medication on one day and no medication on the other day, whereas the control group did not receive any medication. All children with ADHD diagnosis were medicated at the time of the study. The off medication condition had a washout of at least 24 h (up to 2, 5 days). Methylphenidate is rapidly eliminated after intake with a half-life of 3.5 h. Even when using the most long-acting variant Concerta with modified gastro-intestinal release and clinical effect duration of approximately 12 h, almost the entire drug has been eliminated from the blood the next morning (Janssen-Inc, 2013; Katzman and Sternat, 2014). A “wash-out” period of 24 h is therefore good enough for examining the patient in an unmedicated condition. The order of on- and off medication was counterbalanced. Test occasions were also counterbalanced for the TD comparison group in order to control for learning effects between the two test sessions. All tests were conducted between 9 and 11 in the morning to minimize the inconvenience of being off medication.

#### Verbal Episodic Memory Task

Word recall; 5 min (Söderlund et al., 2010). Lists of nouns were presented to the participants on a computer screen using a laptop. Participants were asked to remember as many nouns as possible on each test. Two lists of 12 nouns were presented on each test occasion; the inter-stimulus interval (ISI) was 5 s. Noise and no noise conditions were given in counterbalanced order: six of the words were low frequency words and six high frequency words. Each list was matched for word frequency, word length and syllable number. Immediately after each list, participants were asked to give an oral free recall in any order. Balanced Latin squares were used to ensure that each word list was equally likely to be heard in each noise condition, and within each list words were presented in a random order to each child.

#### Visuo-Spatial Working Memory Task

Spanboard; 5 min (Westerberg et al., 2004). Participants were asked to remember the location of dots that appeared in a 4 × 4 grid (16 squares) presented on a computer screen and to recall this sequence using the computer mouse to click the correct grid locations. In the first trial, the array consisted of two dots; the ISI was 3 s, 2250 ms dot exposure, 750 ms pause. On every second successive trial, one dot was added until the participant made an error in both trials on that particular level. The test was performed two times on each occasion, in noise or in silence in counterbalanced order.

#### The Verbal 2-Back Task

n-Back task; 5 min (Strand et al., 2012). Participants were presented with a sequence of 30 words, and the task consisted in indicating when the current word matched the one from two words earlier in the sequence. Each word was presented for 2250 ms with a pause on 2000 ms between the words (ISI 4.25 s). Participants were instructed to press a button each time the same word as 2-back from the sequence was shown again. Response time and number of errors were counted. Four lists of words were presented on each test occasion, two in silence and two in noise.

## Results

A 2 × 2 × 3 mixed ANOVA was conducted with one between subjects factor: *group* (ADHD vs. TDC) and two within subject factors: *noise condition* (no noise vs. noise) and *task* (spanboard; word recall; 2-back). The ANOVA showed a positive main effect of noise [*F*(1,38) = 5.71, *p* = 0.022, η^2^= 0.131]. The interaction between task and noise, i.e., noise affected tasks differently, did not reach significance but indicated a trend [*F*(2,76) = 3.86, *p* = 0.057]; also a trend toward interaction between group and noise was found [*F*(1,38) = 3.10, *p* = 0.086] indicating a larger noise benefit for ADHD children. The between group factor for overall task performance did not reach significance, however, the TDC group performed tasks marginally superior to the ADHD group [*F*(1,38) = 3.71, *p* = 0.062].

A second 2 × 2 × 3 ANOVA was conducted, just comprising the ADHD group with three within subjects factors: *noise* (no noise vs. noise), *medication* (off vs. on medication), and *task* (spanboard, word recall, 2-back). This ANOVA showed a strong positive main effect of noise [*F*(1,19) = 16.64, *p* = 0.001, η^2^= 0.467] and no main effect of medication [*F*(1,19) = 0.63, *p* = 0.436]. Further, an interaction between medication and noise, i.e., the benefit of noise was larger than the one of medication [*F*(1) = 4.88, *p* = 0.040, η^2^= 0.204], and also an interaction between task and noise was found, noise affected the three tasks differently [*F*(2,18) = 11.40, *p* = 0.003, η^2^= 0.375]. The three-way interaction between task, noise, and medication did not reach significance [*F*(2) = 3.13, *p* = 0.093].

**FIGURE 1:**
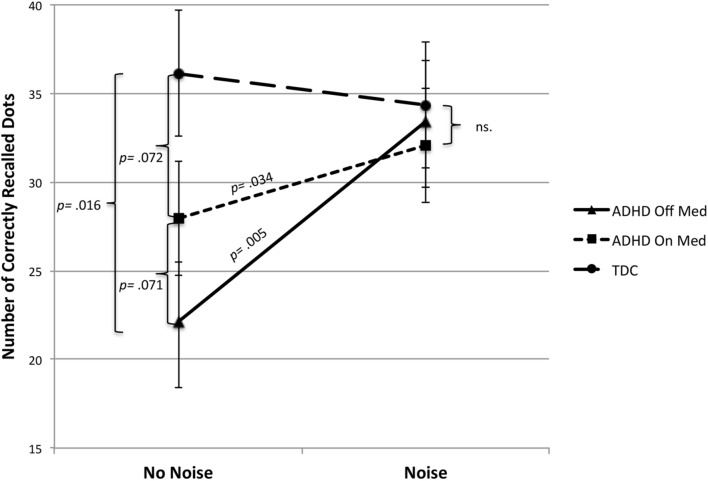
**Number of correctly recalled dots on a visuo-spatial task as a function of medication and noise in ADHD.** As a comparison the performance of TDC is shown as a function of noise; test occasion is counterbalanced for the TDC group. White noise 79–80 dB; TDC, typically developed children; *p*-values are written in the graph for significant and trend significant differences.

*Post hoc* analyses, by using independent samples *t*-tests task by task, showed noise benefit in the spanboard- and word recall task but not in the 2-back task. In the spanboard task a paired samples *t*-test showed that performance in terms of successfully recalled dots increased significantly by introduction of noise as compared to no noise in non-medicated ADHD children [*t* = (19) = 3.18, *p* = 0.005], as well as in medicated children [*t* = (19) = 2.28, *p* = 0.034]. A (2 × 2) within subject ANOVA showed a tendency to interaction between noise and medication [*F*(1,19) = 3.51, *p* = 0.077] in this task (see **Figure 1**).

The control group was not influenced by noise, an independent samples *t*-test displayed that the TDC superior performance in the spanboard task compared to the ADHD group in the silent condition [*t*(38) = 2.52, *p* = 0.016] disappeared during noise exposure [*t*(38) = 0.68, *p* = 0.504]. The medicated children did not fully reach the performance of TDC in no noise condition [*t*(38) = 1.85, *p* = 0.072]. There was no significant effect of medication on children with ADHD in no noise condition, the improvement by medication did not reach significance [*t*(19) = 1.91, *p* = 0.071]. However, the control group did not differ significantly from the ADHD group in the presence of noise irrespective of medication or not (see **Figure 1**).

**FIGURE 2:**
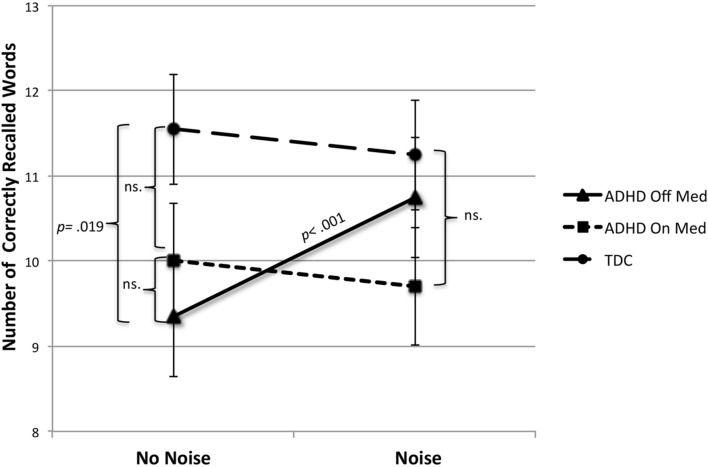
**Number of correctly recalled words as a function of medication and noise in ADHD.** As a comparison the performance of TDC is shown as a function of noise; test occasion is counterbalanced for the TDC group. White noise 79–80 dB; TDC, typically developed children; *p*-values are written in the graph for significant differences.

In the word recall task (free recall), a paired samples test showed that noise significantly improved the number of correctly recalled words compared to no noise condition in absence of medication [*t* = (19) = 4.92, *p* < 0.0005]. In the no noise condition, the non-medicated ADHD group performed worse than the TD group [*t*(38) = 2.45, *p* = 0.019]; however, this difference was eliminated by noise. No effect of medication was found in this task and no effect of noise was found in the ADHD group during medication, or in the control group. Finally we found a significant interaction between noise and medication in this task within the ADHD group [*F*(1,19) = 5.43, *p* = 0.031] (see **Figure 2**). **Table 2** reports performance in raw score in the spanbord- and the word recall tasks for both ADHD- and TDC groups with *t*-statistics.

**Table 2:** Results on Word Recall and Spanboard task for ADHD and control children (TDC) in noise and in silence.

Task/Group	*N*	No noise (SD)	Noise (SD)	Noise vs. no noise *t*-statistics, (*p*)
**Word recall**
ADHD off med	20	9.35 (3.2)	10.75 (3.2)	*t*(19) = 4.21, *p* < 0.001
ADHD on med	20	10.00 (3.1)	9.70 (3.1)	ns.
Off vs. On med	20	ns.	ns	–

Control (TDC)	20	11.55 (2.9)	11.25 (3.7)	ns.

**Spanboard**
ADHD off med	20	22.15 (11.0)	33.45 (16.2)	*t*(19) = 3.18, *p* = 0.005
ADHD on med	20	27.95 (12.6)	32.05 (14.6)	*t*(19) = 2.28, *p* = 0.034
Off vs. On med	20	0.071	ns.	–

Control (TDC)	20	36.13 (19.3)	34.35 (18.5)	ns.

*ADHD children were medicated on one test occasion, in counterbalanced order*.

In the verbal 2-back task no significant results of either noise or medication was found in either group (see **Table 3**). When looking at the number of correctly recalled items both groups performed equally well [ADHD = 22,5 vs. TDC = 22,9; F(1,38) = 0.07, *p* = 0.787]. No interaction between group and noise and no main effect of noise on ADHD was found (*p* = 0.136). No effect of medication was found. There was a nominal positive effect of noise on the ADHD group but it was far from reaching any significance. However, when looking at the response time we got a significant group difference, as TDC were faster than the ADHD group [34.1 vs. 40.8 s; *t*(38) = 2.81, *p* = 0.008]. The only effect of noise exposure was on the TDC group where noise increased speed on task performance [35.3 vs. 32.8; *t*(19) = 2.17, *p* = 0.043]. Noise had little effect on the speed of the ADHD group and the interaction between noise and group indicated a trend [*F*(1,38) = 3.35, *p* = 0.075]. At last, the effect of test occasion was non-significant, but nominally there was a slight improvement for the TDC group in the second test session.

**Table 3:** Results on verbal 2-back task for ADHD and control children in noise and in silence. Number of correct responses and response times are displayed.

Task /Group	*N*	No noise (SD)	Noise (SD)	Noise vs. no noise *t*-statistics
**2-back task, number correct**				
ADHD off med	20	22.2 (3.9)	23.0 (3.3)	ns.
ADHD on med	20	21.8 (4.6)	22.9 (3.8)	ns.

Control	20	23.1 (6.8)	22.8	ns.

**2-back task, response time in sec.**				
ADHD off med	20	40.4 (7.6)	40.1 (8.3)	ns.
ADHD on med	20	40.1 (11.6)	42.8 (8.6)	ns.

Control	20	35.3 (8.8)	32.8 (11.2)	ns.

*ADHD children were medicated on one test occasion, in counterbalanced order*.

## Discussion

Indeed the present study revealed a substantial effect of noise exposure on cognitive task performance but interestingly no significant effects of stimulant medication. A significant interaction between noise and medication was found where the noise benefit was larger than the effect of medication. Again, this confirms earlier findings of small or no effects of medication on cognitive task performance (Hazel-Fernandez, 2004; Hellwig-Brida et al., 2011) and that different tasks demand different dosages depending on task requirements to yield positive effects (Berman et al., 1999; Evans et al., 2001). These results show that, unlike medication, the introduction of auditory noise considerably improves task performance in inattentive children. Although previous studies have shown beneficial effects of noise on cognition in ADHD (Söderlund et al., 2007), this is the first study making a direct comparison between noise therapy and medication. Furthermore, the combination of noise and medication did not add anything further to performance implying that there is no beneficial effect of combining the two treatments. Of note is that in the word recall task medication destroyed the noise effect and worsened performance as compared with just noise. This could indicate that the combination of medication and noise pushed performance over the top as indicated by the inverted U-curve in SR (Sikström and Söderlund, 2007). On the other hand this was not obvious in the Spanboard task, and the interactions between noise and medication need to be investigated further. However, both noise and medication eliminated the differences in cognitive performance between the ADHD and the TDC groups. The noise effect was present in both an executive- (spanboard) and a non-executive memory task (word recall), which indicates that noise can be helpful in a broad range of skills that are important for school performance. Looking at our hypotheses more specifically results showed the following: firstly, a superior performance for TD children in all tasks; secondly, a noise improvement for the ADHD group but no effect of noise on performance for the TD group; thirdly, no significant improvement of medication for the ADHD group; fourthly, an interaction between noise and medication indicating that noise was more efficient than medication and that the combination of noise and medication did not add anything further to performance. This lack of medication effects on cognition is indeed interesting; as mentioned in the background the effects of medication on cognitive and school performance are not evident. The reasons for this can be several; one possibility is that medication levels for optimal effect differs considerably between individuals, which could result in inappropriate levels (Vitiello et al., 2001; Huss et al., 2014). Another possibility is that the evaluation of medication effects, like in the Huss et al. (2014) study, only uses DSM related outcome measures as evaluation of medication. No studies have, to the best of our knowledge, systematically explored the outcomes of different medication levels on a cognitive test battery; this calls for further investigation.

The nil finding in the 2-back task is in this context somewhat puzzling, as there was no effect either of noise or of medication in this task. Group differences in the 2-back task between TDC and medication naïve ADHD patients are found in both reaction time and accuracy (Bayerl et al., 2010). In n-back task workload that can be manipulated by both difficulty (0, 1, 2, 3 back) and pace (inter-stimulus-intervals, ISI), we used the 2-back task with a constant ISI in the present study. When manipulating workload one study found no effects of medication in 0-back or 2-back task, but found some in the 3-back task in accuracy and no effects on reaction time in any of the three conditions (Kobel et al., 2009). The opposite, a medication effect, has however been found in the 2-back task in both reaction time and accuracy (Ramasubbu et al., 2012). Even under very low workload conditions (0-,1-,2-back) improvements due to medication are found (Strand et al., 2012). But interestingly in this study, the improvement by medication was not more substantial than that resulting from providing incentives to participants, i.e., gaining points for correct answers that could be exchanged for toys, games or gift cards (Strand et al., 2012). From this it can be concluded that incentives (or motivation) have to be taken into account when comparing results from different studies. Yet another study showed significant effects of medication on reaction time (faster) but no effect on accuracy in any of the tasks: 0-,1-,2-back (Tomasi et al., 2011). Nevertheless, our study displays a significant group difference in the 2-back task in response time between TDC and ADHD children, although not in accuracy. Moreover, only controls were influenced by the noise exposure and became faster, but the ADHD group did not change reaction times in any direction. Thus, factors like workload, mental state, and inter-stimulus-intervals have to be taken to account.

From our results it is suggested that noise can be introduced into the neural system through the auditory modality, and spread to regions not necessarily coding for sounds, as there are examples of cross modal noise benefit in both children and adults (Manjarrez et al., 2007; Helps et al., 2014; Baijot et al., 2016). Earlier research has shown that the addition of noise to a signal can improve the detection of the signal that is required to pass a certain threshold, a phenomenon called SR (see Moss et al., 2004 for a review). Moreover, when the signal is sufficiently high, then noise is detrimental to discrimination and the relation between noise level and performance will show an inverted U-curve (Sikström and Söderlund, 2007). This was, however, not supported in the present study where noise did not have any influence on performance of the TD group. An earlier study using three noise levels did not succeed either to show an inverted U-curve in any group, control or ADHD (Helps et al., 2014). This may indicate that there are different patterns in threshold SR compared to supra threshold SR that act on cognitive performance (McDonnell et al., 2007).

The present findings could possibly support the suggestion that the neural noise level is associated with a low dopamine tone in inattentive children and that noise may increase target saliency through the phenomenon of SR, thus improving cognitive performance (Sikström and Söderlund, 2007). Although acoustic noise is not found to increase dopamine levels *per se*, it looks like external noise in the nervous system acts in a similar fashion as dopamine release, indicated by findings from a rat model of ADHD (Pålsson et al., 2011). As further support for the SR view a recent study showed neurophysiological effects on EEG/ERP of noise exposure during a GoNogo task where the P300 signal increased in the children that did benefit from noise (Baijot et al., 2016). Furthermore, a recent fMRI study found increased activity in dopaminergic areas of the brain in healthy adult controls exposed to noise during cognitive task performance (Rausch et al., 2014). Future research has to use brain-imaging techniques to investigate the mechanisms of noise benefit further.

As an alternative explanation, noise benefit might be understood in relation to the so-called underarousal and regulation of vigilance theories of ADHD. According to these theories, individuals with ADHD often have low activity in important neural circuits in the brain, and thus need external stimuli and frequent reward to regulate and increase the neural activity above the threshold in order to be able to concentrate better when performing assignments (Satterfield and Dawson, 1971; Barry et al., 2009; Clarke et al., 2013; Federspiel et al., 2013; Geissler et al., 2014; Mayer et al., 2015). According to the vigilance regulation model (Hegerl et al., 2010), individuals with ADHD or mania have an unstable vigilance regulation with compensatory hyperactivity leading to increased arousal. This is also in line with the state regulation deficit model of ADHD (Sonuga-Barke et al., 2010) that is derived from the cognitive energetic theory (Sergeant, 2005). This theory posits that ADHD patients have difficulties in modulating and maintaining their arousal levels, particularly in boring tasks, and that hyperactivity in this light would be regarded as a homeostatic response. However, there are few studies in which arousal is experimentally manipulated in ADHD, while arousal can be defined in different ways: quantitative EEG, alpha, beta, gamma-waves (Buyck and Wiersema, 2015), event rate (Sonuga-Barke et al., 1992; Kooistra et al., 2010), or direct physical measures as heart rate and blood pressure (Börger and van der Meere, 2000). However, when assessing energetic levels, indirect measures like event rate and work load are used as indicators where slow rates or low load are claimed to produce under-arousal, and fast rates and high load produce over-arousal (Sergeant, 2005). The problematic part is that behavioral performance is taken as a proxy for arousal state where poor performance is regarded as an indicator of state regulation deficits without providing any physiological measures as supporting evidence.

Another strong candidate for the cause of noise benefit is auditory masking, where a masker different from the signal can facilitate signal detection (Durlach et al., 2003). In ADHD It has been shown that if the masker is predictable children behave less impulsively (Gray et al., 2002). Moreover, masking effects have been shown in both visual and tactile modalities (Tan et al., 2003; Dawes et al., 2009). One possible way to determine the role of masking in noise benefit is to use stochastic vestibular noise (SVS) that seems to have the same or a similar effect on performance as auditory noise (Wilkinson et al., 2008, 2009; Samoudi et al., 2014).

### Limitations

We are fully aware that this is just a pilot study and not even close to a randomized control trial study (RCT). The design is quasi experimental and treatments are not blinded or double blinded; in particular, noise is for obvious reasons impossible to blind. We are at this moment running a blinded noise study using stochastic vestibular electric noise (SVS), as this technique allows both participants and experimenters to be blinded. In such a setting, one conducts a proper randomized trial control study to compare the effects of noise with medication while simultaneously controlling the effect of auditory masking. Another obvious limitation to the present study is the small number of participants.

## Conclusion

In the present pilot study we show that exposure to 80 dB of auditory white background noise can offer a possible non-pharmacological, alternative or complementary treatment to medication when treating inattention in school children.

## Author Contributions

GS planned and designed the experiment, performed the statistical analysis and drafted the manuscript. CB designed the experiment and was responsible for the data collection. PG have been involved in revising the manuscript critically for important intellectual content. All authors read and approved the final manuscript.

## Conflict of Interest Statement

The authors declare that the research was conducted in the absence of any commercial or financial relationships that could be construed as a potential conflict of interest.
